# Microwave‐Assisted Subcritical Water Extraction Method for the Optimized Isolation of Cellulose From Residual Soybean Hulls

**DOI:** 10.1002/cssc.71012

**Published:** 2026-08-26

**Authors:** Monica Rigoletto, Maria Laura Tummino, Elisa Natali, Chiara Angileri, Giorgio Grillo, Silvia Tabasso, Veronica Bortolotto, Claudia L. Bianchi, Giancarlo Cravotto, Enzo Laurenti

**Affiliations:** ^1^ Department of Chemistry University of Turin Turin Italy; ^2^ Institute of Intelligent Industrial Technologies and Systems for Advanced Manufacturing (STIIMA) Italian National Research Council (Cnr) Biella Italy; ^3^ Department of Drug Science and Technology University of Turin Turin Italy; ^4^ Department of Chemistry University of Milan Milan Italy

**Keywords:** agro‐industrial waste, cellulose, circular economy, life‐cycle assessment, microwave

## Abstract

Agro‐industrial lignocellulosic wastes represent a huge source of biopolymers, proteins, carbohydrates, and active compounds. Their fractionation through eco‐friendly routes is a challenge in accessing the components of interest while minimizing environmental impacts. In this study, a 20‐min microwave‐assisted subcritical water extraction (MASWE) was selected to isolate cellulose from soybean hulls. This emerging green hydrothermal process, coupled with a microwave‐assisted alkaline (MW‐NaOH) treatment, enabled the reduction of harsh chemical employment compared to classical biomass fractionation, ensuring, at the same time, a high cellulose enrichment of up to 91.5% in the best procedure. The environmental impacts of the up‐scaled optimized strategy were compared to those of the multi‐hydrolysis treatment through a life‐cycle assessment analysis. The MW‐based cellulose extraction provides clear advantages in toxicity‐related categories, but it is still necessary to reduce the energy‐driven impacts.

## Graphical abstract

Almost pure cellulose was extracted from soybean hulls by microwave‐assisted alkaline treatment (MW‐NaOH) and characterized. The microwave‐assisted subcritical water extraction (MASWE) of waste biomass falls within the circular economy approach, promotes sustainability, and reduces both treatment times and the use of harsh chemicals. Scale‐up and life‐cycle assessment (LCA) confirm the advantages of this procedure in cellulose recovery from exhausted biomass.

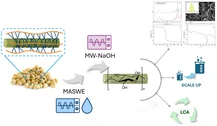

## 1 Introduction

With a global production of around 396 million metric tons in 2023/2024 (United States Department of Agriculture), soy is intensively cultivated on a global scale since it is widely used for the production of food, feed, medical products, and several industrial applications [1].

Soy is considered a highly nutritious crop plant, whose beans present an abundance of proteins (35%–45%), lipids (15%–25%), carbohydrates (30%–35%), dietary fibers (17%), and traces of micronutrients [1, 2, 3]. Furthermore, other interesting components such as isoflavones, saponins, lecithin, phytic acids, glycine, bioactive peptides, and vitamins make soybeans’ nutritional profile well‐known for its health benefits [1, 4, 5]. Hence, its use in human food products and animal feed is widespread. During industrial soy processing, various by‐products including lecithin, soy meal, soy‐whey, hulls, okara, and molasses are produced. Most of them could enter valorization paths and be recovered [1].

An interesting residue for biomass valorization is represented by soybean hulls, which are primarily generated during the extraction of soy oil and in all the processes that need a de‐hulling treatment.

Soybean hulls represent 8% w/w of the seed and are mainly constituted by cellulose (29%–51%), hemicellulose (10%–25%), lignin (1%–18%), pectin (4%–30%), and proteins (11%–15%) [6, 7, 8, 9, 10]. Due to their composition, they are widely used in animal feed as a source of fibers, as well as for human dietary fibers or in the medical field. The lignocellulosic nature of hulls also opens up their application in biorefineries, where they can be converted into fuels or chemical building blocks, transformed into micro and mesoporous adsorbents, or extracted to recover polysaccharides such as cellulose [1, 11].

Cellulose is an unbranched linear polymer composed of D‐glucose repeating units linked by β—1,4‐glycosidic bonds. In plants and, thus, in the residual soybean hulls, these cellulose macromolecular chains are arranged together to form elementary fibrils through the inter‐ and intramolecular hydrogen bonds between the many hydroxyl groups that cellulose possesses [12]. Furthermore, in such a lignocellulosic matrix, hemicellulose covers the surface of the cellulose fibrils, held together by noncovalent bonds, and increases the affinity toward lignin, thereby favoring the cohesion between the three components [13]. These interactions have to be taken into account when considering the isolation of one of the components.

Cellulose has many interesting properties, including abundant reserves, degradability, biocompatibility, a large specific surface area, thermal stability, and adjustable rheological properties [12, 14]. Furthermore, due to its large number of active —OH groups, it allows the development of various derivatives with designable properties via oxidation, esterification, etherification, grafting, and cross‐linking [15, 16, 17]. Its ease of functionalization makes cellulose an interesting basis for the development of a wide range of materials that can find applications in the food industry, packaging, batteries, drug delivery, cosmetics, or water treatments [18, 19]. Moreover, recently, the interest in waste‐derived cellulose (i.e., recovered from agricultural scraps, industrial by‐products, and even waste textiles) is increasing to lower the environmental impact related to the use of virgin plants [13].

The fractionation of lignocellulosic biomass can be achieved through both alkaline and acid hydrolysis of existing bonds binding cellulose, hemicellulose, and lignin [20, 21, 22]. In the alkaline environment, the fibers swell because of the break‐up of the hydrogen bonds between the cellulose chains, and the cleavage of the bonds within hemicellulose and lignin occurs. On the other hand, acid treatments ensure, among other things, hemicellulose solubilization and depolymerization. The combination of all these effects leads to the release of the cellulosic components [6].

Despite the efficiency of these techniques in fractionating lignocellulosic biomasses, it is necessary to use strong acids and bases. According to the Green Chemistry principles [23], the safety of reagents and solvents is a central challenge, and more and more often, the choice falls on water. Indeed, water is the most environmentally friendly solvent as it is nonflammable, nontoxic, and readily available. However, its dielectric properties limit its solvent power to polar molecules only. These properties can be modified under particular temperature and pressure conditions below the critical point (373.946 °C, 22.064 MPa), reaching the so‐called subcritical conditions, in which it can be maintained in the liquid form even above the normal boiling point [24, 25, 26, 27].

Generally, in the range between 100 and 374 °C, as the temperature rises, the dielectric constant of water (*ε*′  =  80 at 25 °C) decreases, reaching values near those of organic solvents and increasing its solvent capacity of low‐ and medium‐polarity compounds [24, 27, 28].

Furthermore, subcritical water has a slightly acidic nature thanks to the self‐ionization that provides more hydronium ions (H_3_O^+^) that facilitate acid‐catalyzed reactions [29, 30, 31].

According to the literature, many studies employed this technique to extract bioactive compounds [32, 33], pectin [34], proteins, lipids [35], sugars [36], nutraceuticals, and antioxidants from food and herbaceous plants [37, 38]. However, there are a limited number of studies using subcritical water in processes to obtain cellulose from biomass [39, 40].

The mildly acidic nature of subcritical water selectively hydrolyzes hemicelluloses without degrading the cellulosic moieties [30]. During the hydrothermal process, hemicellulose deacetylation occurs, leading to the release of acetic acid with a consequent decrease in pH, which can, in turn, catalyze the hydrolysis of hemicelluloses to shorter oligomers and, consequently, decrease their molar mass [41, 42, 43]. At the same time, the breaking of ether and ester bonds that bind hemicellulose and lignin takes place, also leading to the release of the lignin fraction [44, 45].

Subcritical water treatments are generally used as biomass pretreatments capable of altering or modifying the lignocellulosic matrix in a way that provides easier access to chemical reagents or enzymes for subsequent hydrolysis [46, 47]. Subcritical conditions can be achieved through a hybrid technique involving rapid microwave (MW) heating under controlled temperature and pressure‐sealed vessels in which water is heated above its normal boiling point [48, 49].

In this study, we select this green technique coupled with a second microwave‐assisted alkaline treatment to isolate fibrous cellulose from a soybean hull sample that has already been deprived of the enzyme soybean peroxidase (SBP) [50]. The extraction process has been optimized, and all the cellulose‐enriched biomasses obtained have been characterized as well, to find the best compromise between isolation efficiency (in terms of cellulose purity) and impact. Indeed, we compared the impacts of the optimized extraction with a multi‐hydrolysis procedure previously developed by our research groups [50, 51]. To this aim, a scale‐up study was conducted, and a life‐cycle assessment (LCA) was performed to define a possible paradigm for feasible, green, and efficient cellulose extraction from residual biomass.

## 2 Experimental Section

### 2.1 Materials

All reagents were used without purification. Fresh yellow soybean (*Glycine max*) seeds were purchased from Del Prete s.r.l. (Fondi, LT, Italy). Sodium hydroxide (NaOH), gallic acid, sodium carbonate (Na_2_CO_3_), Folin–Ciocalteu reagent, ethanol, benzene, and sulfuric acid were purchased from Sigma–Aldrich.

### 2.2 Microwave‐Assisted Subcritical Water Extraction (MASWE) Experiments

Soybean hulls employed for cellulose isolation were previously subjected to SBP extraction, dried, and ground. Then, the hulls (approx. 1 g for the screening and 60 g for the scale‐up) were mixed with distilled water at a 1:10 solid–liquid ratio in a Teflon vessel, left to moisturize for 5 min, and introduced into a MW multimodal reactor (SynthWAVE, Milestone, Bergamo, Italy) able to exploit an external inert gas feeding (N_2_). For each test, appropriate purging with N_2_ was performed three times to remove oxygen traces from the system, reducing oxidative stress on the biomass. The reaction chamber was then pressurized with the necessary amount of N_2_ to avoid water ebullition (20 bar). The samples were heated at different temperatures (150, 180, and 200 °C) with a maximum irradiation power of 1500 W. The temperature was increased through a 10‐min ramp and maintained constant for an additional 10 min under stirring.

The resulting solutions were filtered under vacuum while thoroughly washing the biomass with distilled water. Both the liquid and the washed biomass were freeze‐dried (LyoQuest‐85, Telstar, Madrid, Spain), weighed, and stored at 4 °C for further analyses. Three cellulose‐enriched samples were obtained on the basis of the extraction temperature: MW‐SWE‐150, MW‐SWE‐180, and MW‐SWE‐200.

The extraction results (Biomass %, Extract %, and Loss %) are calculated through Equations (1), (2), and (3), respectively.

(1) $$\text{Biomass}\;\%=\frac{\text{Biomass after MASWE}\;(g)\;}{\mathrm{Raw}\;\text{biomass}\;(g)}\times 100$$

(2) $$\text{Extract}\;\%=\frac{\mathrm{Dry}\;\text{extract}\;(g)}{\mathrm{Raw}\;\text{biomass}\;(g)}\;\times 100$$

(3) $$\mathrm{Loss}\;\%=\frac{\left(\text{Theoretical recovery}\;\left(g\right))-(\text{biomass after MASWE}\;(g)\right)}{\text{Theoretical recovery}\;(g)}\times 100$$

The theoretical recovery was calculated by the difference between the total mass of the sample before MASWE treatment and the mass of the freeze‐dried MASWE extract.

### 2.3 Microwave‐Assisted Alkaline (MW‐NaOH) Treatment

The freeze‐dried biomasses were also subjected to a microwave‐assisted alkaline treatment to remove any residues of hemicellulose and lignin.

A proper amount of biomass (approx. 1 g for the screening and 60 g for the scale‐up) was mixed with a NaOH solution (10% w/w) at a 1:20 solid–liquid ratio in a Teflon vessel and then introduced into the MW multimodal reactor. For this treatment, the samples were heated at 120 °C with a maximum irradiation power of 1500 W. The temperature was increased through a 10‐min ramp and maintained constant for 120 min under stirring.

The resulting solutions were filtered under vacuum, neutralized, and freeze‐dried. The biomass was washed and neutralized before freeze‐drying and storage. Three samples were obtained, labeled MW‐SWE‐150‐A, MW‐SWE‐180‐A, and MW‐SWE‐200‐A, where 150, 180, and 200 indicate the temperature of the previous subcritical water extraction, and “A” indicates the alkaline treatment.

### 2.4 Biomass Physical‐Chemical Characterization

Morphological investigations were conducted with an EVO 10 scanning electron microscope (SEM, Carl Zeiss AG, Oberkochen, Germany), setting an acceleration voltage of 20 kV. The samples were sputter‐coated with a gold layer of 20 nm in rarefied argon using a Quorum Q150R ES Plus Sputter Coater. The attenuated total reflectance Fourier transform infrared (ATR‐FTIR) spectra were recorded using a Spectrum Two UATR (PerkinElmer) instrument in the range of 4000–600 cm^−1^ to evaluate the vibrational features of the materials.

The isolated biomasses were also investigated for their thermal behavior by thermogravimetric analysis (TGA) and differential scanning calorimetry (DSC). For TGA analyses (TGA 1 Star System of Mettler Toledo, Schwerzenbach, Switzerland), about 10 mg of sample was heated from room temperature to 100 °C, left at this temperature for 30 min, and then heated to 800 °C at a rate of 10 °C/min in 30 mL/min of nitrogen flux. The first derivative of TGA (DTG) was obtained from the main thermogravimetric curve to find the main phenomena associated with mass loss. DSC of samples was carried out with a calorimeter Mettler Toledo 821e (Schwerzenbach, Switzerland), calibrated by an indium standard. The calorimeter cell was flushed with 100 mL/min N_2_. The run was performed from 30 to 500 °C, at a heating rate of 10 °C/min, and the sample mass was about 5 mg. Data were processed through the embedded STARe Software.

X‐ray diffraction (XRD) analyses were performed with a Rigaku Miniflex 600 diffractometer equipped with a multistrip detector (128). Cu Kα radiation was employed, and the following operating parameters were set: 40 kV, 15 mA, and 0.02° step size. The obtained XRD patterns have been compared with reference 00‐003‐0289 from the ICDD/JCPDS database relative to a native cellulose Iβ/Iα mixed structure with a prevalence of monoclinic cellulose Iβ. The crystallinity index (C.I.%) was calculated using both the Segal and standard area methods. In the first, the C.I.% is calculated from the height ratio between the intensity of the crystalline peak (*I*_002_–*I*_AM_) and the total intensity (*I*_002_), according to the existing literature [52] Equation (4) was used:

(4) $$C.I.{\%}_{\text{Segal}}=\frac{I_{002}-I_{\mathrm{AM}}}{I_{002}}\times 100$$

where *I*_002_ corresponds to the intensity of the (002) peak, which includes both crystalline and amorphous contributions, whereas *I*_AM_ is the minimum intensity between the (10‐1) and (002) peaks, representing the amorphous fraction [53].

For the standard area method, the area of the crystalline domain (*S*_c_) and the area of the domain in the range 10°–30° 2θ (*S*_t_) are considered, and Equation (5) was used [54].

(5) $$C.I.{\%}_{\mathrm{area}}=\frac{S_{c}}{S_{t}}\times 100$$

X’Pert High Score and OriginPro 8.5 software were employed to treat the data.

### 2.5 Compositional Characterizations

The dried samples were fractionated into their constituents by using different solvents according to the NREL methodology (see Equations S1, S2, S3, and S4) [55].

The optimized process was more deeply characterized to investigate the presence of other valuable fractions. Pectin and lignin precipitation and total phenolic content analysis (TPC, Folin–Ciocalteu assay) have been performed. The procedures and results are described in detail in Supporting Information (see also SEM images of lignin in Figure S1).

### 2.6 Scale‐up

The whole process of MW‐assisted extraction of cellulose from soybean hulls has been optimized using reduced volumes of 10 mL for each extraction. The most promising conditions were reproduced with a volume of 600 mL, which represents a 60‐fold up‐scaling.

### 2.7 LCA

**FIGURE 1:**
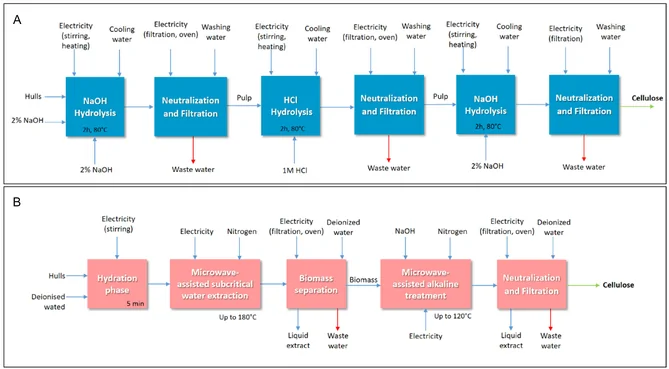
Stages of (A) multi‐hydrolysis cellulose extraction; (B) optimized MW‐assisted cellulose extraction.

A LCA analysis was done to compare the impacts of the scale‐up conditions optimized in this study and the multi‐hydrolysis extraction previously developed by our research groups [50]. LCA provides a standardized framework for quantifying the potential environmental impacts associated with products, processes, or services across their life cycle by systematically accounting for resource use and emissions within defined system boundaries. This study has been designed as a “cradle‐to‐gate” study, and the functional unit was set at 20 g of the output product, i.e., cellulose‐enriched biomass. The boundary of the considered system and various stages are depicted in Figure 1. The LCA model was built in SimaPro 9.6.0.1 software, and the impact assessment was carried out using the CML‐IA baseline method. In line with ISO 14040/14044, the inventory was compiled by combining primary data obtained from the laboratory procedure developed in this study with secondary data taken from the Ecoinvent 3.10 database (cut‐off system model).

#### 2.7.1 Inventory Data Acquisition

Data collection is a critical step in LCA because data quality directly influences the reliability of the results. In this study, primary data were obtained from laboratory measurements, and information was gathered on the quantities of raw materials used, the amount and type of energy consumed, and the outputs generated during the process.

Secondary data were sourced primarily from the Ecoinvent 3.10 database. Due to the limited availability of specific information on transportation, inputs were represented using market datasets in Ecoinvent, which reflect average European supply chains and material availability. For background processes such as raw material production, transport, and waste treatment, datasets were selected following a geographical hierarchy: Italian datasets when available, otherwise European datasets, and global datasets only when no regional alternatives were present.

#### 2.7.2 Impact Assessment

In LCA, the impact assessment phase converts the quantified inventory of elementary flows (resource use and emissions) into environmental indicators by applying characterization factors defined by a selected method. In this study, an impact assessment was performed to compare the environmental burdens of the scale‐up conditions optimized herein with those of the previously developed multi‐hydrolysis extraction. Calculations were conducted in SimaPro 9.6 using the CML‐IA baseline method (Center for Environmental Science, Leiden University). In addition, climate change impacts were evaluated using the IPCC 2021 GWP100 v1.03, which partitions the total GWP100 into fossil, biogenic, and land‐transformation contributions.

Results are reported per functional unit (20 g of cellulose‐enriched biomass) within a cradle‐to‐gate system boundary. In accordance with the Ecoinvent 3.10 cut‐off system model, soybean hulls were treated as a residual by‐product of soybean processing and were therefore included in the system without upstream agricultural or processing burdens. The extraction generates additional fractions (lignin‐rich stream, pectin‐rich stream, and phenolic extracts), but these were not considered coproducts in the present assessment. No allocation or system expansion was applied, and no credits were assigned. All impacts were thus attributed to the production of 20 g of cellulose‐enriched biomass, ensuring a conservative and methodologically consistent comparison between the two processing routes.

## 3 Results and Discussion

### 3.1 Cellulose Extraction and Samples Characterization

**FIGURE 2:**
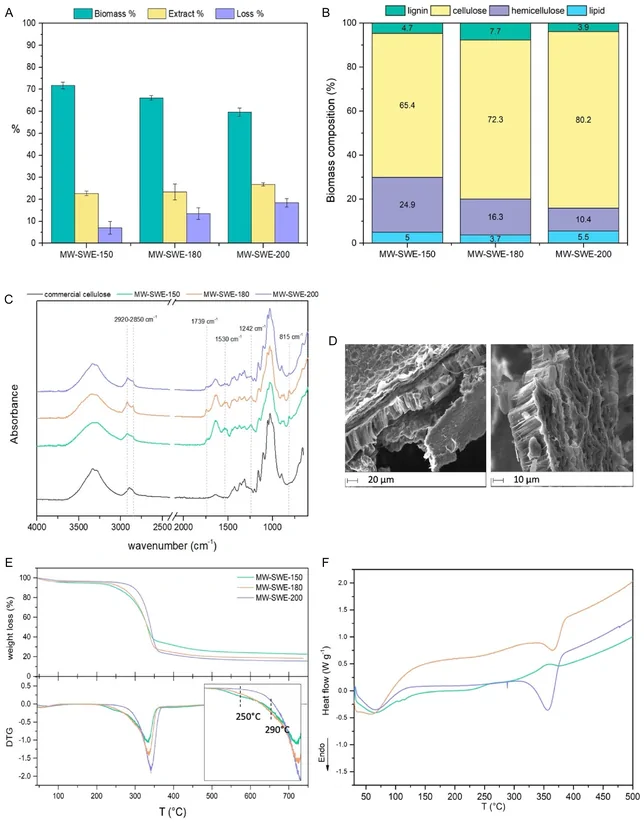
(A) Recovery percentages of biomasses and extracts after MASWE extraction; (B) Outcomes of biomasses’ compositional analysis (NREL) normalized on 1 g of biomass; (C) Comparison between ATR‐FTIR spectrum of commercial cellulose and MASWE derived samples; (D) SEM images of MW‐SWE‐150 (left) and MW‐SWE‐180 (right); (E) TGA and DTG of MASWE samples; (F) DSC of MASWE samples.

After MASWE treatments, three samples of dried residual biomass have been obtained depending on the extraction temperature: MW‐SWE‐150, MW‐SWE‐180, and MW‐SWE‐200. As emerges from Figure 2A, increasing the extraction temperature from 150 to 200 °C led to a reduction in the percentage of solid biomass recovered and an increase in the amount of the extract. The observed increase in biomass loss could be related to a higher formation of low‐molecular‐weight and volatile thermal degradation products during extraction.

Each sample of biomass was subjected to the NREL protocol to investigate the change in percentual composition induced by SWE (Figure 2B). To provide a complete overview of the samples, the pristine soybean hulls and the hulls resulting from SBP extraction were also analyzed (Figure S2). Although the treatment to extract the SBP enzyme reduces the percentage of hemicellulose in the hulls compared to the original biomass, the quantity of cellulose remains practically unchanged at around 41% by weight.

The MASWE extraction leads to cellulose enrichment in all samples with a growing trend depending on the temperature: 65.4%, 72.8%, and 76.6% for MW‐SWE‐150, MW‐SWE‐180, and MW‐SWE‐200, respectively. These increases are accompanied by a loss in lignin, comparable across the three treatments, and a loss in hemicellulose, increasing with the extraction temperature. This trend is in accordance with literature studies [56, 57].

According to Yedro and coworkers [43], the extraction yields of hemicellulose are strongly dependent on temperature. However, as this parameter and extraction time increase, not only does the carbohydrate solubilization increase but also its deacetylation and degradation into oligomers and molecules with lower molecular weight. Hemicellulose deacetylation leads to acetic acid release, which reduces the pH of the solution and catalyzes biomass acid hydrolysis [41, 42]. This causes the breaking of ether and ester bonds that bind hemicellulose and lignin, leading to the release of the latter fraction [44, 45]. Furthermore, the increased acidity of the reaction could promote the formation of degradation products.

ATR‐FTIR spectroscopy and morphological analysis were carried out to investigate the quality of the treated biomasses. As evidenced in Figure 2C, the MASWE extraction is not sufficient to ensure the complete removal of noncellulosic components from the hulls. Indeed, in all sample spectra, there are signals not ascribable to cellulose. The sharp peaks at 2920–2850 cm^−1^ are one of the main differences. Despite a band present in cellulose itself, according to some authors, the emerged intensification of these signals would be ascribable to the asymmetric and symmetric stretching of the methyl and methylene groups that can be found in both lignin [58, 59, 60, 61] and hemicellulose [62]. However, some studies also suggest their possible attribution to the extractives’ content in the biomass matrix, like methyl esters and phenolic acid methyl esters, which contain methyl and methylene groups [60, 63].

Furthermore, the peaks at 1739 and 1242 cm^−1^ are other evidence of hemicellulose residues. According to the literature, the first is the stretching of the C=O present in acetyl and carboxyl hemicellulose functionalities, whereas the second may be related to the C—O stretching in carboxylic groups [64, 65, 66]. On the other hand, the signal around 1530 cm^−1^ could be attributed to the stretching of the C=C contained in the aromatic rings of lignin, while the peak at 815 cm^−1^ to the out‐of‐plane vibration of the guaiacyl unit characteristic of this same lignocellulosic component [64, 67, 68, 69, 70].

SEM images also evidence the presence of noncellulosic residues. Indeed, in the MW‐SWE‐150 and MW‐SWE‐180 samples (Figures 2D and S3A,C), the fibers appear well‐organized and held together by a sort of coating of hemicellulose and lignin. At the same time, although MW‐SWE‐200 presents more separated fibers (Figure S3E) thanks to the stronger extraction temperature, it is still possible to see more aggregated areas, confirming also in this case the presence of noncellulosic residues. Figure S3B,D show some fibers that appear “exploded”. This could be due to the formation of hot spots and the rapid increase in pressure in preferential points within the biomass during the MW treatment [71, 72, 73].

Hemicellulose and lignin lead to a thermal behavior that differs from that of pure cellulose. Figure 2E and Table S1 show that MW‐SWE‐150 and MW‐SWE‐180 samples present thermal degradation temperatures, corresponding to 20% and 50% weight losses, lower than those observed for commercial fibrous cellulose. According to some authors, this could be due to residues of hemicellulose, which begin to decompose earlier than the other lignocellulosic components at a temperature of around 160 °C and whose degradation mainly occurs in the range of 200–300 °C [74]. Two characteristic weight loss peaks have been reported: (i) at 250 °C due to the cleavage of glycosidic bonds and the decomposition of side chains and (ii) at 290 °C due to depolymerization reactions [74, 75, 76, 77]. The appearance of two shoulders on the main peak in correspondence with these temperatures in the experimental DTG curves of both MW‐SWE‐150 and MW‐SWE‐180 (Figure 2E inset) could confirm the presence of hemicellulose impurities in the samples. This evidence agrees with the noncellulosic signals in the ATR‐FTIR pattern (Figure 2C) and the fibers’ morphology, as evidenced by SEM images (Figure 2D). Conversely, MW‐SWE‐200 thermally behaves more similarly to commercial cellulose (Table S1), confirming a greater removal of noncellulosic components, as already emerged with NREL analysis (Figure 2B) and SEM images (Figure S3E).

By decreasing the SWE treatment temperature from 200 to 150 °C, the residual char in the TGA analysis increases (15.5% for MW‐SWE‐200, 18.2% for MW‐SWE‐180, and 22.6% for MW‐SWE‐150). This trend is opposite to the amount of cellulose calculated through NREL analysis, which is higher as treatment temperatures rise. Therefore, it can be assumed that higher TGA residues correspond to higher hemicellulose and lignin content since these biopolymers tend to form more char. This statement is corroborated by several studies related to the thermal behavior of the different lignocellulosic constituents, as discussed hereafter.

Cellulose thermal degradation occurs mainly through the fusion of the crystalline portion together with depolymerization and subsequent rapid degradation reactions that form volatile compounds, which are mostly condensable organic molecules [75]. Conversely, even if hemicellulose pyrolysis also goes through depolymerization, it leads to the formation of less volatile compounds and a greater quantity of char [78, 79, 80, 81]. Indeed, according to some authors, the macromolecular fragments resulting from the early stages of degradation cannot be stabilized by dehydration, thus evolving toward repolymerization reactions and generating char [82].

Lignin significantly influences the amount of char as well. As reported in several studies, during thermal degradation, it does not undergo depolymerization but still releases volatile compounds, which are mainly noncondensable gases. This appears to be due to the instability of the propyl chains as well as some linkages between monomer units and the methoxy substituents of the aromatic rings. The final lignin charring process involves the rearrangement of the carbon‐based skeleton into a polycyclic aromatic structure [75, 83, 84].

All the samples described so far resulted in higher TGA residues (at 750 °C) than commercial fibrous cellulose (8%) and soy‐derived cellulose obtained by adopting the multi‐hydrolysis method (13.8%) described by Tummino et al. [50]. Regarding the DSC curves (Figure 2F), only MW‐SWE‐200 exhibits a pattern comparable to that of commercial cellulose, with an endothermic peak at the decomposition temperature of 356 °C [50]. No endothermic peaks are detected in MW‐SWE‐150, while MW‐SWE‐180 shows an intermediate behavior.

**FIGURE 3:**
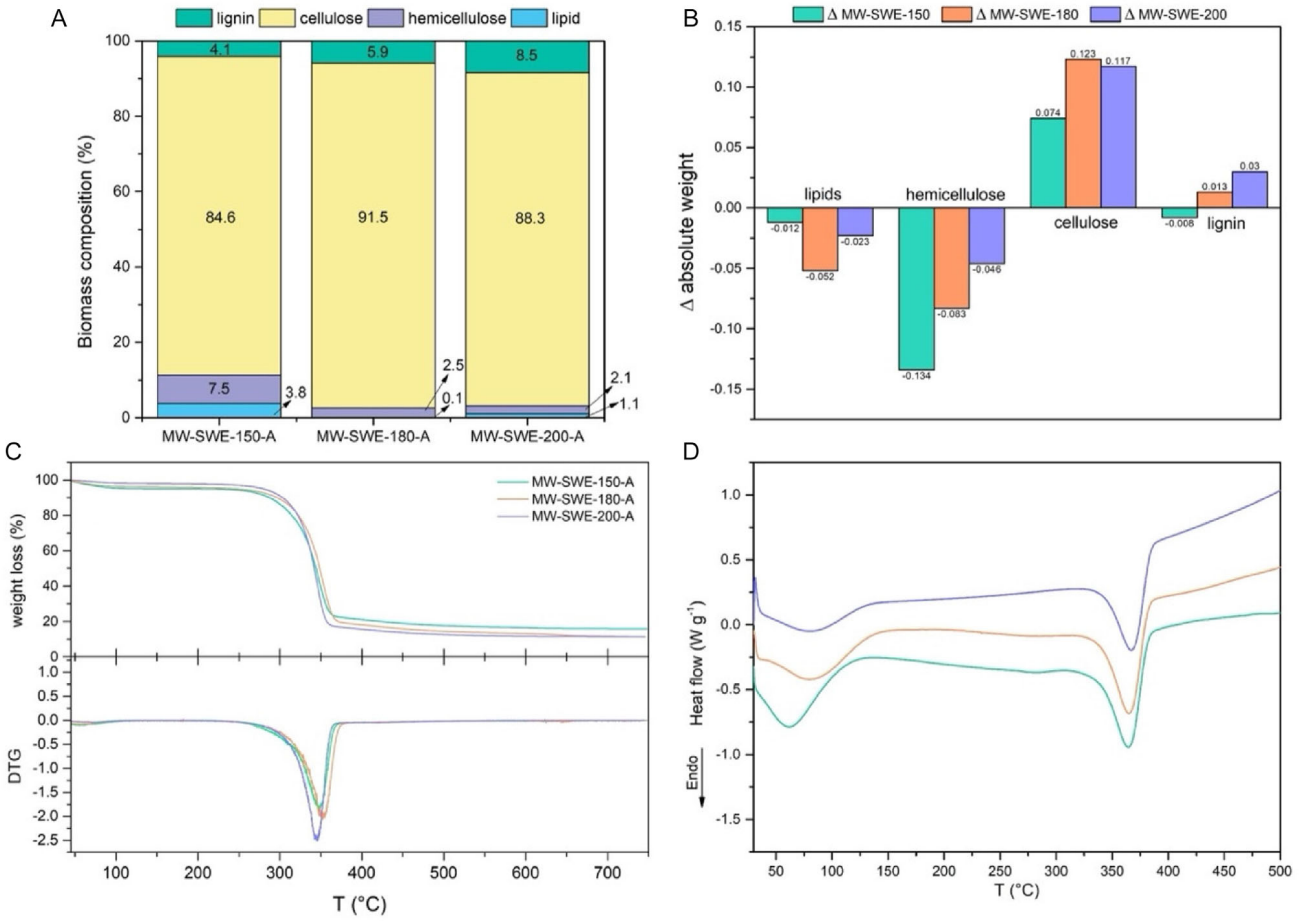
(A) Outcomes of biomasses’ compositional analysis (NREL) normalized on 1 g of biomass; (B) Enrichments and losses of the components—calculated as absolute weight differences—after MW‐NaOH; (C) TGA and DTG of samples after alkaline treatment; (D) DSC of samples after alkaline treatment.

Given these results, after the MASWE treatment, all samples were subjected to MW‐assisted alkaline extraction (MW‐NaOH) to complete the removal of hemicellulose and lignin and to enhance cellulose enrichment. As shown in Figure 3A, the enrichment in cellulose induced by the alkaline treatment is effective, with the best results for MW‐SWE‐180‐A (91.5%). This is mainly due to a reduction in the hemicellulose content, as highlighted by Figure 3B, which depicts the differences in the absolute weight of each component in the three resulting biomass samples. Indeed, in alkaline media, cellulose swells, and the hydrogen bonds with hemicellulose are disrupted. At the same time, alkali‐labile ester bonds between lignin monomers or lignin and hemicellulose are broken. Both of these mechanisms enhance the dissolution of hemicellulose [85, 86]. The quality of the cellulose‐enriched biomasses has been investigated through ATR‐FTIR and morphological analysis. As shown in Figure S4, the ATR‐FTIR pattern of all the samples is comparable to that of commercial cellulose, confirming cellulose as the main component.

**FIGURE 4:**
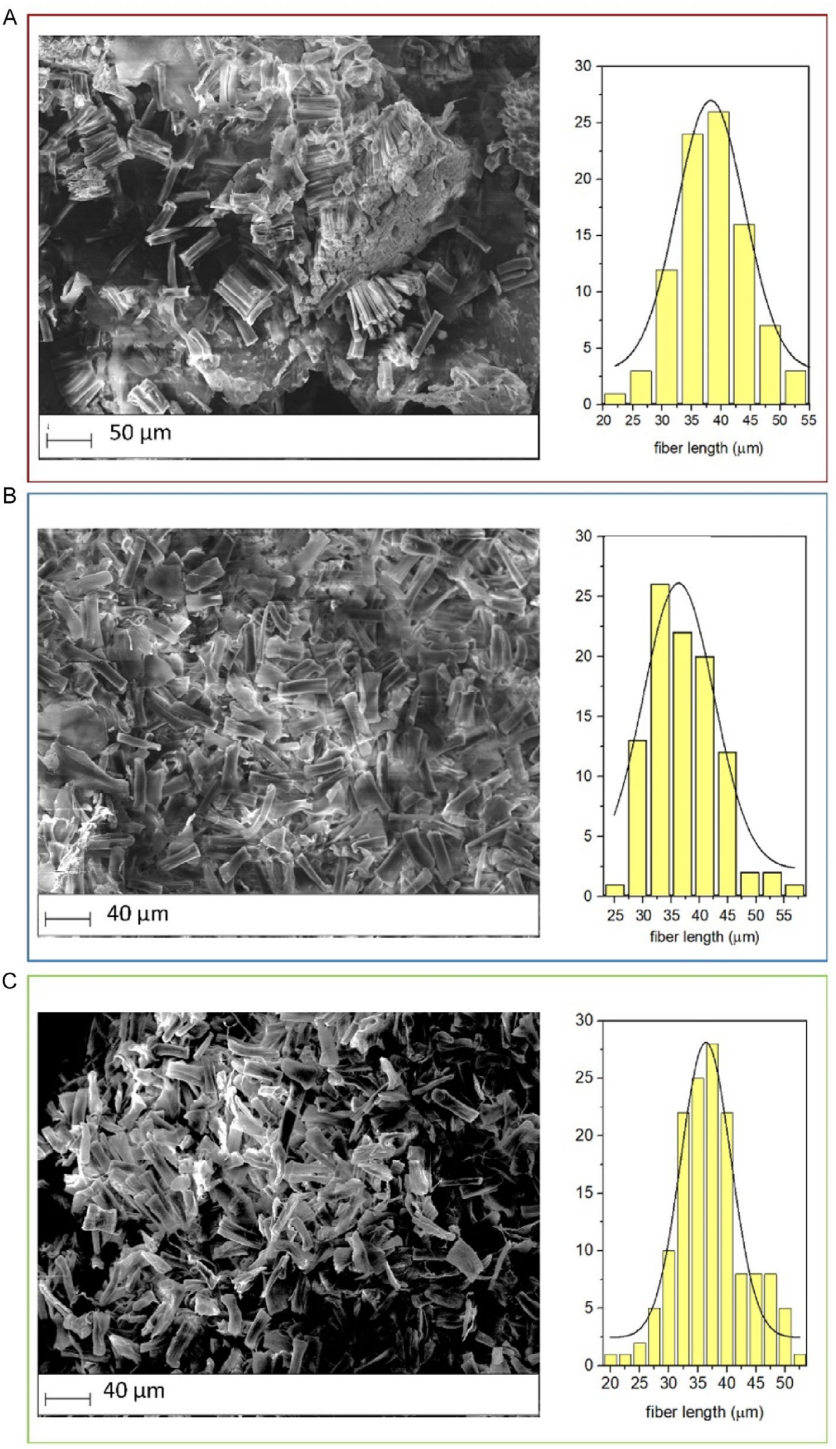
SEM images (left) and fiber length distribution (right) of (A) MW‐SWE‐150‐A, (B) MW‐SWE‐180‐A, and (C) MW‐SWE‐200‐A.

Despite this, the morphological analysis highlighted that for MW‐SWE‐150‐A, even the alkaline treatment was insufficient to completely separate the fibers (Figure 4A). Conversely, MW‐SWE‐180‐A (Figure 4B) and MW‐SWE‐200‐A (Figure 4C) morphologies are characterized by well‐separated fibers with a measured average length between 35 and 40 µm (Figure 4A–C), shorter than commercial cellulose (60 µm) but comparable with that of cellulose derived from the hulls multi‐hydrolysis process [50].

The final biomass thermal behavior is summarized in Figure 3C,D and Table S1. The thermal degradation temperatures corresponding to 20% and 50% weight losses of all the samples are close to those of commercial cellulose, while the DTG endothermic peaks occur at lower heating temperatures, particularly for MW‐SWE‐150‐A. This sample also presents a shoulder on the main peak at 290 °C in the DTG graph, suggesting the presence of limited residues of hemicellulose. This is also evident by observing the MW‐SWE‐150‐A morphology, which shows cellulose fibers still stuck together (Figure 4A). On the contrary, considering both the DTG endothermic peak temperature (Figure 3C and Table S1) and samples' morphology (Figure 4B,C), it can be stated that MW‐SWE‐180‐A and MW‐SWE‐200‐A have features comparable to multi‐hydrolysis cellulose, confirming a similar extraction treatment efficiency. However, as shown by the TGA and DTG data (Table S1), MW‐SWE‐200‐A shows a slight loss in thermal stability compared to the other samples, suggesting that the processing conditions for cellulose isolation in this case are too harsh.

These effects can be derived from the strong interaction of MW, resulting in the weakening of the hydrogen‐bond network within the molecular cellulose matrix at temperatures higher than 180 °C, which allows the polar CH_2_OH groups to act similarly to “molecular radiators” [87]. From all these considerations, it can be stated that the isolation of cellulose through MASWE at 180 °C, followed by alkaline extraction, represents the best option.

This is also supported by XRD results of the cellulose‐enriched biomasses, which are reported in Figure S5, together with the peak deconvolution results and the native cellulose reference diffractogram (00‐003‐0289). All samples showed the typical diffraction peaks of cellulose I, indicating that the combined MASWE and alkaline treatment preserved the native cellulose structure.

The calculated crystallinity indices (C.I.%_Segal_, Table S2) were consistent with the literature values reported for soybean hull‐derived cellulose (~70%) [8, 10]. Although the different subcritical water extraction conditions had a limited overall effect on the crystalline structure, the rise in the extraction temperature progressively reduced the intensity of the main crystalline peak (002) and decreased the C.I.%_Segal_ from 75.6% for MW‐SWE‐150 to 69.2% for MW‐SWE‐200. A comparable trend has been found by calculating the crystalline index with the standard area method (C.I.%_Area_, Table S2). This suggests that increasing treatment severity progressively affects not only amorphous regions but also ordered cellulose domains.

One cause can be found in the accessibility of cellulose fibers to NaOH, and SEM observations support this interpretation. The MW‐SWE‐150 sample showed compact and sticky fibers linked by residual noncellulosic components, whereas MW‐SWE‐180 and MW‐SWE‐200 exhibited more separated fibers with lower amounts of hemicellulose and lignin. The residual matrix in MW‐SWE‐150 may have partially limited NaOH accessibility, thereby preserving the cellulose crystalline domains and resulting in a higher crystallinity index despite the lower cellulose purity.

Conversely, the more severe pretreatments at 180 and 200 °C enhanced fiber separation and facilitated NaOH penetration, improving cellulose recovery but also increasing the disruption of the hydrogen bond network and partially decreasing the crystalline order. Therefore, excessively mild SWE conditions were insufficient to achieve high cellulose purity, whereas overly strong treatments had negative effects on cellulose crystallinity.

Similar behaviors were reported by Osei‐Bonsu et al. [88], who attributed the decrease in crystallinity at higher temperatures to excessive hydrolysis and partial disruption of cellulose nanocrystals, and by Oriez et al. [86], who observed weakening of the cellulose hydrogen‐bond network above 180 °C. Likewise, Peng et al. [89] reported that the combination of microwave irradiation and NaOH treatment can reduce cellulose crystallinity.

Considering the combined results from XRD, SEM, and the other characterization techniques, MW‐SWE‐180‐A appears to provide the best compromise between cellulose recovery, purity, and preservation of the crystalline structure.

### 3.2 Scale‐up Procedure

The MW‐SWE‐180‐A extraction procedure was then scaled up.

**FIGURE 5:**
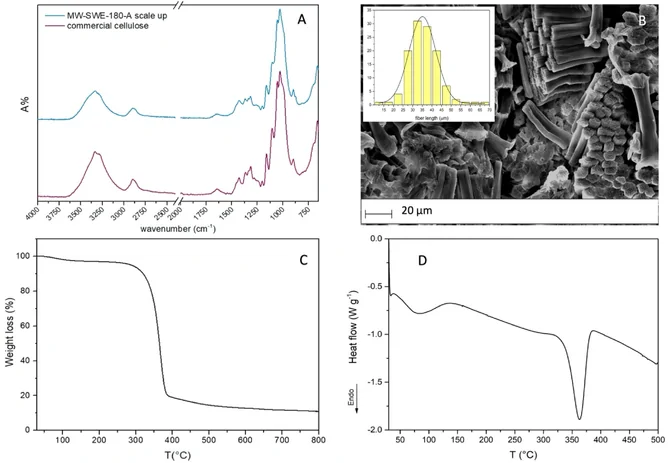
MW‐SWE‐180‐A scale‐up characterizations: (A) ATR‐FTIR; (B) SEM images and fiber length distribution (inset); (C) TGA and (D) DSC curves.

The ATR‐FTIR analysis confirms the effectiveness of noncellulosic components’ removal, and this is consistent with the spectrum of commercial cellulose (Figure 5A).

From a morphological point of view (Figure 5B), the scaled‐up MW‐SWE‐180‐A presented fairly separated fibers comparable with those obtained in smaller volumes and with an average length of 35 µm (Figure 5B—inset). As evidenced in Figure 5C,D, its thermal behavior was comparable to that of MW‐SWE‐180‐A, and the efficiency of the extraction can be further highlighted by examining the limited TGA char residue (10.9 %).

XRD diffractogram of the scaled‐up sample has been recorded in order to study its crystallinity and compare it with the MW‐SWE‐180‐A sample. As shown in Figure S6, the biomass obtained from the scale‐up treatment exhibited the same characteristic diffraction peaks of cellulose I, indicating that the increase in processing volume does not affect biopolymer crystallinity significantly.

The calculated crystallinity index was comparable to that of the corresponding unscaled sample, showing only a slight increase (Table S2), meaning that the overall crystalline organization of cellulose was preserved after scale‐up. However, the main crystalline peaks (002) and (040) resulted in apparently higher intensities and slight broadening than the equivalent unscaled sample (Table S2), while the overall background intensity also increased.

According to the existing literature, peak broadening in cellulose diffractograms may be associated with enhanced structural disorder due to the presence of a greater amorphous fraction in the final biomass or to nonuniform strain within the structure. Amorphous components, lacking long‐range periodic order, can generate diffuse X‐ray scattering over a broad angular range and the appearance of a broad halo, which induces the rise of the background of the entire diffractogram [52, 90]. Nevertheless, the overall crystallinity parameters show that the cellulose crystalline structure was preserved after the scale‐up. Therefore, the moderate differences observed in the diffractograms are likely associated with minor variations in sample organization and intrinsic biomass heterogeneity rather than with significant changes in the crystalline structure of cellulose. Furthermore, the configuration of the MW reactor allows flexibility in treating sample amounts (1–60 g) within the same 1 L liner, so the supplied radiation and its uniformity do not change. The preservation of the cellulose I diffraction pattern, together with the results of thermal analysis, ATR‐FTIR spectroscopy, and fiber length measurements, confirms the effectiveness of cellulose extraction from soybean hulls under the optimized scale‐up conditions.

Given these outcomes, this product was considered optimal from the physical and chemical characteristics’ point of view.

### 3.3 LCA

**FIGURE 6:**
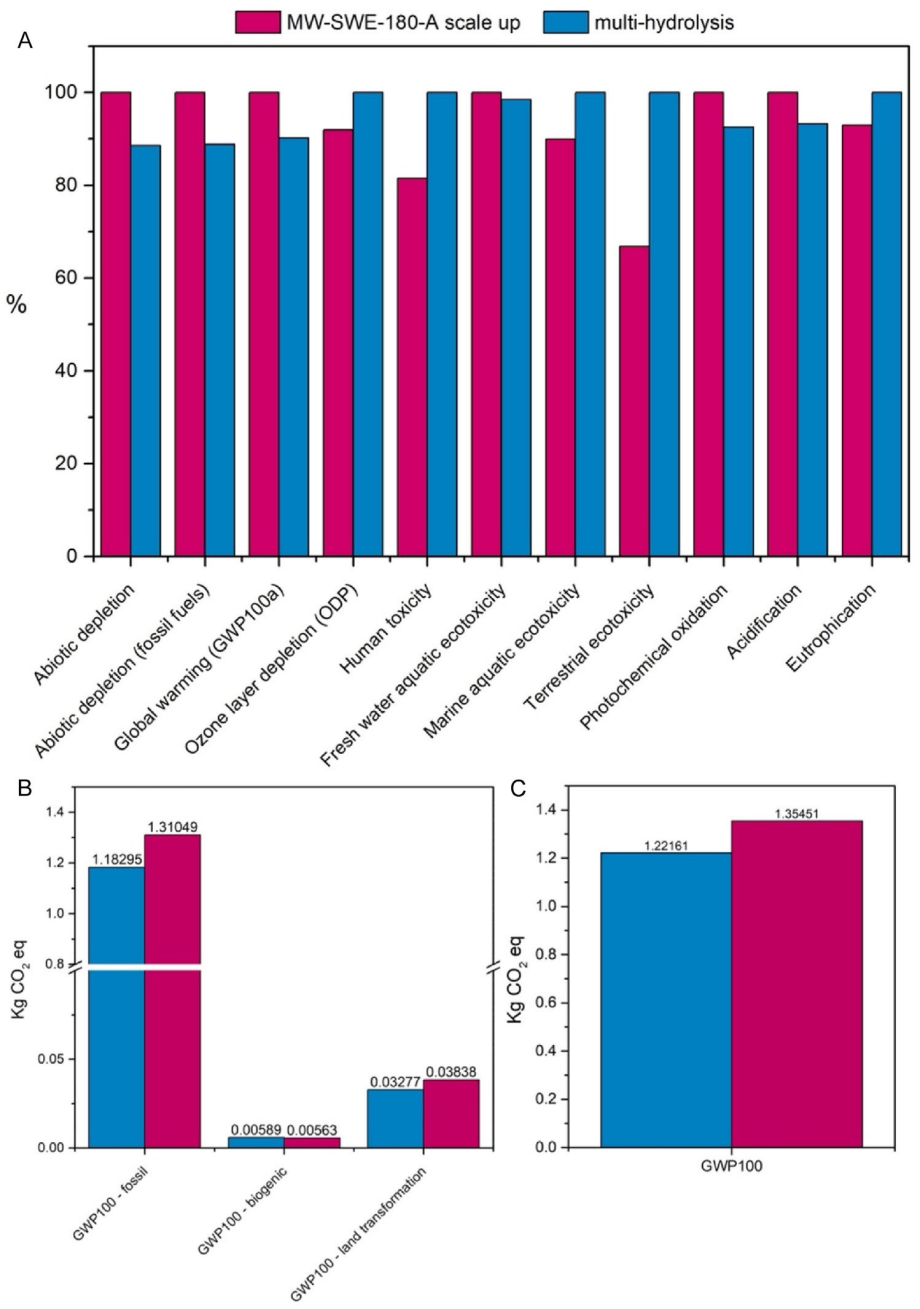
LCA results: (A) CML‐IA baseline V3,10/EU25 method—Characterization; (B) IPCC 2021 GWP100 V1,03 method characterization; (C) IPCC 2021 GWP100 V1,03 method Breakdown of GWP100. Error bars are not reported as the LCA results are based on deterministic calculations rather than statistical replicates.

These promising results led to the deepening of the material impact. An LCA study was conducted to compare the up‐scaled procedure and the multi‐hydrolysis procedure previously developed by our research group. The CML‐IA baseline results (Figure 6A and Table S3) show that the two methods have overall comparable environmental profiles, with most impacts in the same order of magnitude. The largest between‐method differences appear in terrestrial ecotoxicity, human toxicity, and ozone‐layer depletion.

For the multi‐hydrolysis method, higher terrestrial ecotoxicity and human toxicity are primarily associated with electricity consumption and the use of tap water for reflux/cooling in each step, which carries upstream burdens from the water‐distribution infrastructure (e.g., cast‐iron piping, pumping, and treatment) that propagate into these toxicity indicators. In ozone‐layer depletion, a notable contribution arises from hydrochloric acid usage because the background datasets from Ecoinvent include upstream chlorodifluoromethane supply chains; even minute fugitive emissions in those chains have very high ODP characterization factors, thereby amplifying this category. In contrast, the MW‐SWE‐180‐A process shows higher values in energy‐linked categories (e.g., ADP fossil and GWP100a) because electricity supply is the dominant hotspot in both systems and is larger for the MW method due to high‐temperature microwave‐assisted processing.

In addition to the CML‐IA analysis, Figure 6B,C report the climate‐change results obtained using the IPCC GWP100 (2021) method. The total global warming impact remains within the same order of magnitude for both methods, although the MW‐SWE‐180‐A scale‐up shows a slightly higher footprint (1,35 kg CO_2_‐eq) compared with the multi‐hydrolysis method (1,22 kg CO_2_‐eq). This difference is primarily related to the fossil CO_2_ contribution, which dominates the overall category and reflects the greater electricity demand associated with the high‐temperature microwave‐assisted steps. Biogenic carbon and land transformation contributions are minimal for both systems. Overall, the results confirm that the climate impacts of the two methods are very similar in magnitude, with energy consumption remaining the main driver.

Nevertheless, the performance of the MW‐SWE‐180‐A process is promising, and targeted scale‐up optimization could further reduce its footprint, especially in energy‐linked categories, without compromising the advantages observed in toxicity‐related indicators.

### 3.4 Comparative Analysis of Extraction Methods

Against the backdrop of increasing international interest in sustainable cellulose extraction technologies, the proposed method was compared with existing approaches reported in the literature (Table S4).

Overall, the outcomes of the most widespread methods highlight that cellulose extraction from lignocellulosic biomass is strongly influenced by both the intrinsic composition of the raw material and the severity and combination of the applied treatments. Processes based on subcritical water, often coupled with alkaline steps and multiple H_2_O_2_ bleaching cycles, represent a well‐established strategy for the selective removal of hemicellulose and lignin. However, these approaches show significant variability in terms of yield and purity.

Microwave‐assisted processes, even combined with innovative green solvents (NADES) or organosolv processes, emerge as promising alternatives thanks to the reduction of reaction times and, in some cases, the simultaneous improvement of yield and purity. More complex hybrid methodologies, such as steam explosion followed by microwave‐assisted alkaline hydrolysis and microfluidization, are effective in producing high‐purity cellulose nanofibers but with lower yields and high energy demand. This approach appears more suited to high‐value‐added production rather than large‐scale recovery. In this context, the approach developed in this work—combining microwave‐assisted subcritical water treatment with a subsequent microwave‐assisted alkaline step—proved effective for soybean hulls, a hemicellulose‐rich and low‐lignin biomass. Under optimized conditions, a cellulose‐enriched fraction was obtained with a yield of 45% and a purity of 91.5%.

Our strategy provides a good balance between yield and purity while avoiding the need for multiple bleaching steps. Although some fully microwave‐assisted systems reported in the literature (Table S4) achieve higher performances, they often require harsher chemicals or more complex solvents. By contrast, the present method operates under relatively mild alkaline conditions and shorter processing times.

An important observation from the literature survey is that only a limited number of studies include a LCA or even a preliminary energy analysis. The majority of studies did not quantify the electricity consumption, thermal energy demand, or carbon emissions associated with the extraction process. This lack of environmental evaluation represents a gap in the current literature and poses some issues for a critical comparison of our extraction strategy with the existing research. Nevertheless, considering Table S4, we can state that our first step's conditions are comparable in temperature to several studies reported in the literature, where subcritical water treatments are commonly performed between 170 and 180 °C for 30–90 min. Regarding the second step, compared with other alkaline treatments reported in the literature, which often use NaOH concentrations between 2.5 and 5 wt% and temperatures up to 140 °C, the present approach relies on significantly lower alkali concentrations, potentially decreasing chemical consumption and downstream neutralization requirements. The use of microwave assistance may also improve the heat‐transfer efficiency and reduce energy losses compared with conventional conductive heating systems. A direct comparison with a conventional Parr reactor (0.16 L vessel) further highlighted the practical advantages of MW‐assisted heating. When the 180 °C, 120 min treatment was reproduced under conventional conditions, the system reached the target temperature only after 38 min, despite a nominal 10‐min ramp. As a result, the heating step accounted for ca. 31.7% of the isothermal holding time was reduced to only ca. 8.3% in the MW‐assisted process with a 10‐min ramp. This difference can mainly be ascribed to the thermal inertia and wall effects associated with the thick stainless steel structure of the Parr reactor, which is required to withstand autogenous pressure. Conversely, MW dielectric heating enables a much steeper temperature increase through more direct energy transfer to the reaction medium, thereby markedly shortening the nonisothermal phase of the process. This may limit undesired biomass degradation during heating while also reducing the overall operating time as the conventional system required 158 min in total, corresponding to a 21.5% longer process compared with the MW‐assisted treatment. Additionally, and importantly, the energy consumption normalized to the amount of treated biomass was approximately 10% higher in the Parr system than in the MW setup. It is also worth noting that the energy demand of the MW reactor included the chiller unit, which was not present in the conventional configuration. Overall, the optimized conditions proposed in this study appear less chemically intensive and partially less energy‐demanding than many literature approaches, particularly those involving prolonged hydrothermal treatments, repeated bleaching cycles, or high‐pressure mechanical processing.

## 4 Conclusion

A new, sustainable protocol for the isolation of cellulose from soybean hulls was described using a MASWE. These conditions allowed for the avoidance of mineral acids and the reduction of extraction times to 20 min. A further MW‐assisted alkaline extraction (180 °C, 2 h) completed hemicellulose and lignin removal to enhance cellulose enrichment up to 91.5%.

The samples were deeply characterized to elucidate the effect of MW‐assisted treatments on the composition and structures of the solid residues. A scale‐up study as well as a LCA shed light on a new sustainable process for the valorization of soybean hulls according to a biorefinery approach. From an LCA perspective, the MW‐SWE‐180‐A process exhibits an environmental profile broadly comparable to the multi‐hydrolysis route, with most impact categories falling within the same order of magnitude.

The MW‐assisted method provides clear advantages in toxicity‐related categories, whereas energy‐driven impacts remain its main contributors due to the higher electricity demand associated with microwave heating. Overall, the results indicate that the MW‐SWE‐180‐A strategy is environmentally promising, and they point to the potential for further improvements, particularly through energy optimization, to enhance its overall performance.

## Funding

This work was supported by NODES programme, supported by the MUR‐M4C2 1.5 of PNRR funded by the European Union – NExtGenerationEU (ECS00000036).

## Conflicts of Interest

The authors declare no conflicts of interest.

## Supporting information

Additional experimental details on characterization procedures (pectin and lignin precipitation, total phenolic content, NREL, and TGA), Sankey diagrams, and CML‐IA baseline V3,10/EU25 method results for up‐scaled optimized MASWE and multi‐hydrolysis methods are provided.

## Data Availability

The data that support the findings of this study are available from the corresponding author upon reasonable request.
