# Supplementary material for: Microwave‐Assisted Subcritical Water Extraction Method for the Optimized Isolation of Cellulose From Residual Soybean Hulls
Source: ChemSusChem. 2026 Aug 26;19(17):e71012. doi: 10.1002/cssc.71012 (PMC13510119; doi:10.1002/cssc.71012)
Supplement: Supplementary file 1 [file CSSC-19-e71012-s001.pdf]

Microwave-Assisted Subcritical Water Extraction Method for The Optimized Isolation of Cellulose from Residual Soybean Hulls

Monica Rigoletto [a], Maria Laura Tummino [b], Elisa Natali [a], Chiara Angileri [a], Giorgio Grillo [c], Silvia Tabasso [c*], Veronica Bortolotto [d], Claudia L. Bianchi [d], Giancarlo Cravotto [c], Enzo Laurenti [a*]

Supporting Information

Other characterizations' procedures:

Pectin and lignin precipitation [1]**:**

First, 100 mL of the liquor derived from MW-NaOH of the optimized subcritical water treatment were collected and neutralized with HCl 1M to induce pectin precipitation. The solution was left to settle for 24 hours and then centrifuged and filtered in order to separate the supernatant from the solid phase. The solid residue was washed with distilled water to remove traces of soluble salts formed during the neutralization step and dried at 50°C overnight.

The supernatant was acidified up to pH 2 using HCl 1M to precipitate the lignin, which is insoluble in acidic conditions. The solution was left to settle for 72 hours, centrifuged and filtered to separate the precipitate from the supernatant. The solid residue was washed with distilled water up to neutrality, freeze-dried, weighed and morphologically characterized.

Total Phenolic Content (TPC):

The TPC of the freeze-dried liquid fraction deriving from the MASWE treatment of the optimized sample was determined using the Folin–Ciocalteu assay, as described by Cravotto and co-workers [2]. In brief, polyphenol quantification was performed by applying a calibration curve of gallic acid as a reference compound (with dilutions between 5 and 250 µg/mL) in aqueous solutions. Dried extracts were dissolved in deionized water at concentrations of 10, 25 and 50 mg/mL. Both gallic acid and sample solutions (250 µL) were dispensed into borosilicate test tubes. The following solutions were added sequentially for each test: 500 µL of Na_2CO_3 solution (10% w/v), 4 mL of distilled water, and 250 µL of Folin–Ciocalteu reagent (diluted 1:1 with distilled H_2O). The resulting mixtures were vigorously shaken and kept at room temperature for 25 min

before the analysis. A Cary 60 UV-Vis spectrophotometer (Agilent Technologies, Santa Clara, CA, USA) was used to read the absorption at 725 nm in a quartz cuvette (1 cm path length). The TPC was expressed as mg/g of gallic acid equivalents (GAE) over the dried extract (selectivity) and over the dried matrix (yield). The measurements were performed in triplicate.

Results:

The MW-SWE-180-A was selected as the most promising treatment and was therefore subjected to additional characterizations. The neutralization of the final liquor did not lead to the precipitation of a significant amount of pectin. According to the literature, they should represent 4-10% w/w of the total soybean hull composition [3]. This lack of pectin could be ascribed to the subcritical water extraction step, which may have caused its degradation. Indeed, temperatures in the range of 100-250 °C together with acidic conditions could facilitate pectin conversion into galacturonic acid and low-molecular-weight oligosaccharides. 96 mg of lignin have been precipitated, representing ~2% of the biomass before the alkaline treatment. As shown in **Figure S1**, its morphology is characterized by irregular and angular shapes with smooth surfaces. These results are consistent with those reported by Pradyawong et al.[4], who suggest that this appearance can be caused by the alkaline treatment.

TPC assays revealed a total polyphenol concentration of 114 mg GAE per gram of extract.

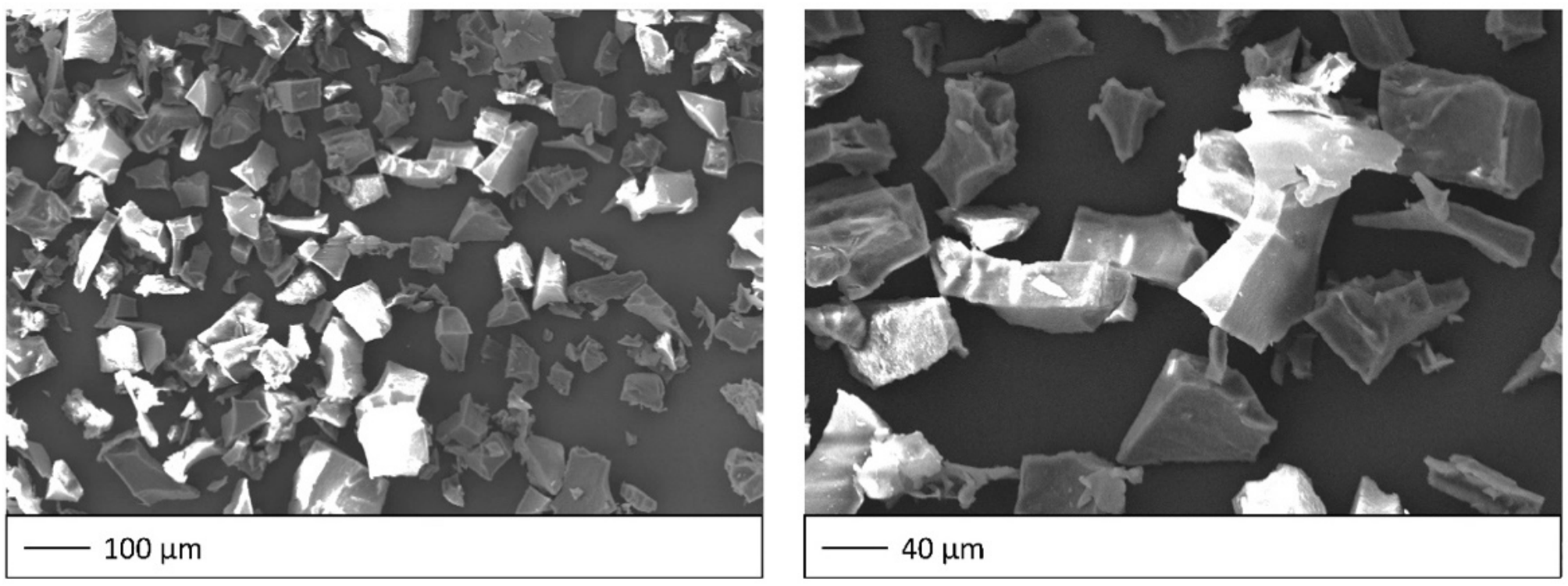

Figure S1. SEM images of MW-SWE-180-A-derived lignin

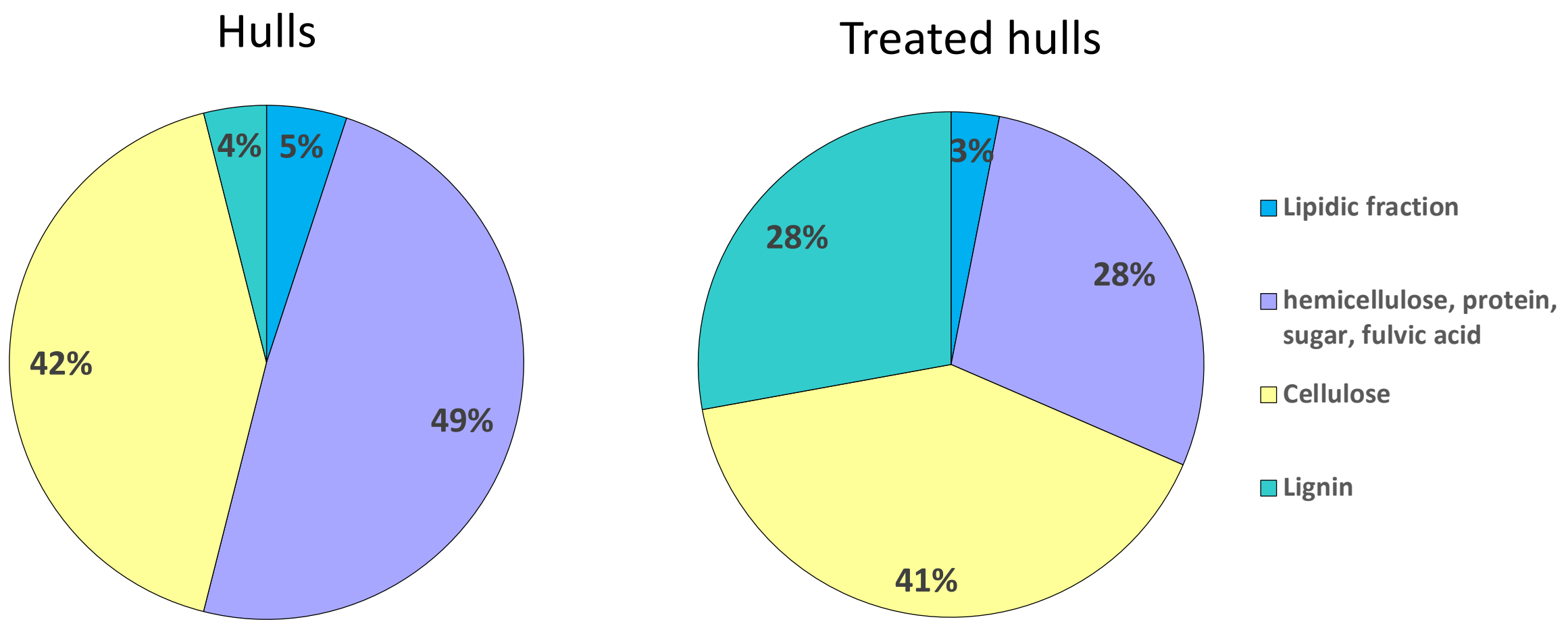

Figure S2: Outcomes of biomass compositional analysis (NREL) of original soybean hulls and treated soybean hulls (namely after soybean peroxidase extraction), normalized on 1g of biomass

These percentages have been calculated using the following equations:

$$Lipids\ \% = \frac{Raw\ Biomass\ (g) - NREL\ Biomass\ I\ (g)}{Raw\ biomass\ (g)} \times 100 \qquad \textbf{(eq. s1)}$$

$$Emicellulose\ \% = \frac{NREL\ Biomass\ I\ (g) - NREL\ Biomass\ II\ (g)}{Raw\ biomass\ (g)} \times 100 \qquad \textbf{(eq. s2)}$$

$$Cellulose\ \% = \frac{NREL\ Biomass\ II\ (g) - NREL\ Biomass\ III\ (g)}{Raw\ biomass\ (g)} \times 100 \qquad \textbf{(eq. s3)}$$

$$Lignin\ \% = \frac{NREL\ Biomass\ III\ (g) - NREL\ Ashes\ (g)}{Raw\ biomass\ (g)} \times 100 \qquad \textbf{(eq. s4)}$$

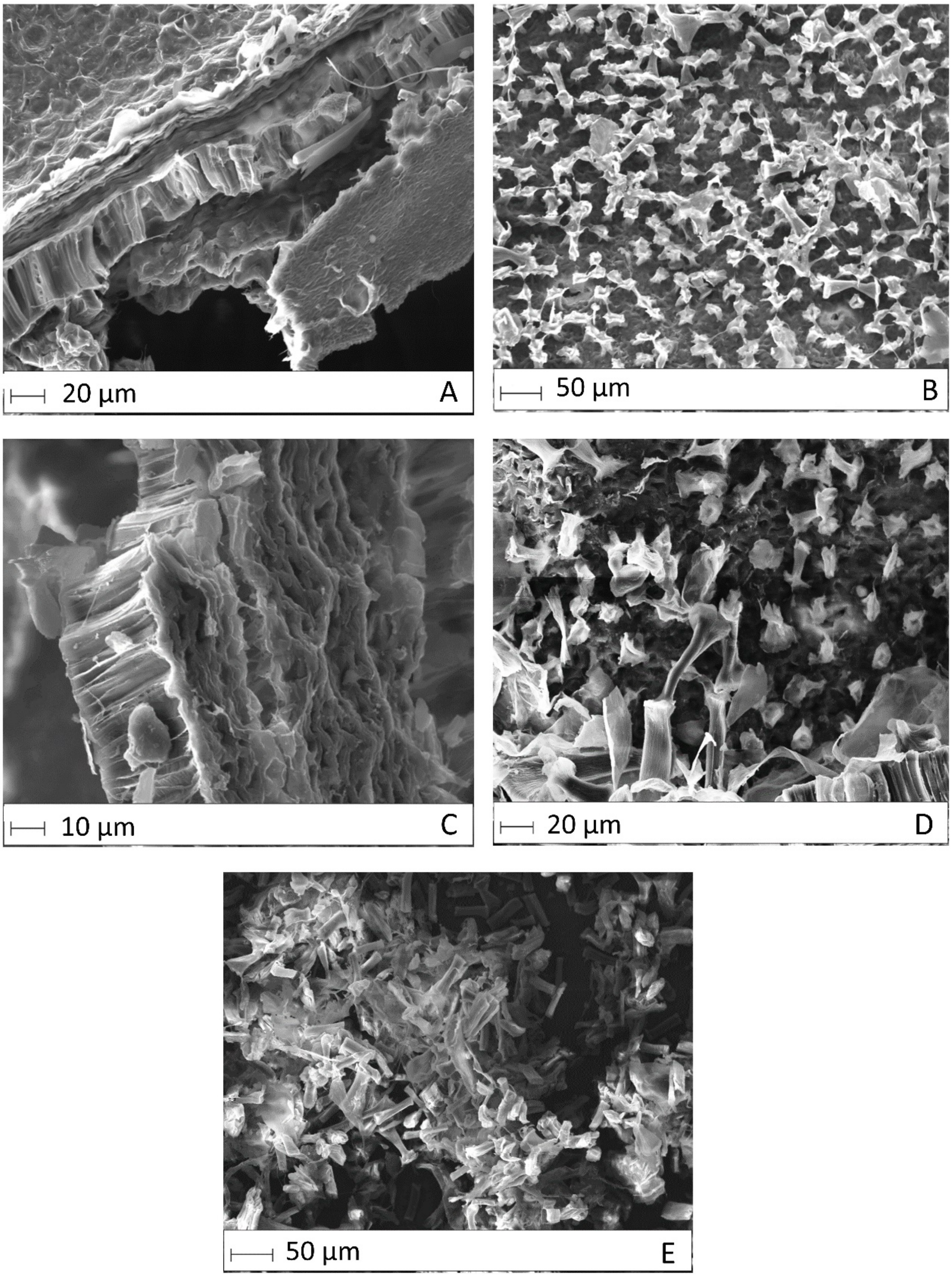

Figure S3. SEM images of MW-SWE-150 (A and B), MW-SWE-180 (C and D) and MW-SWE-200 (E)

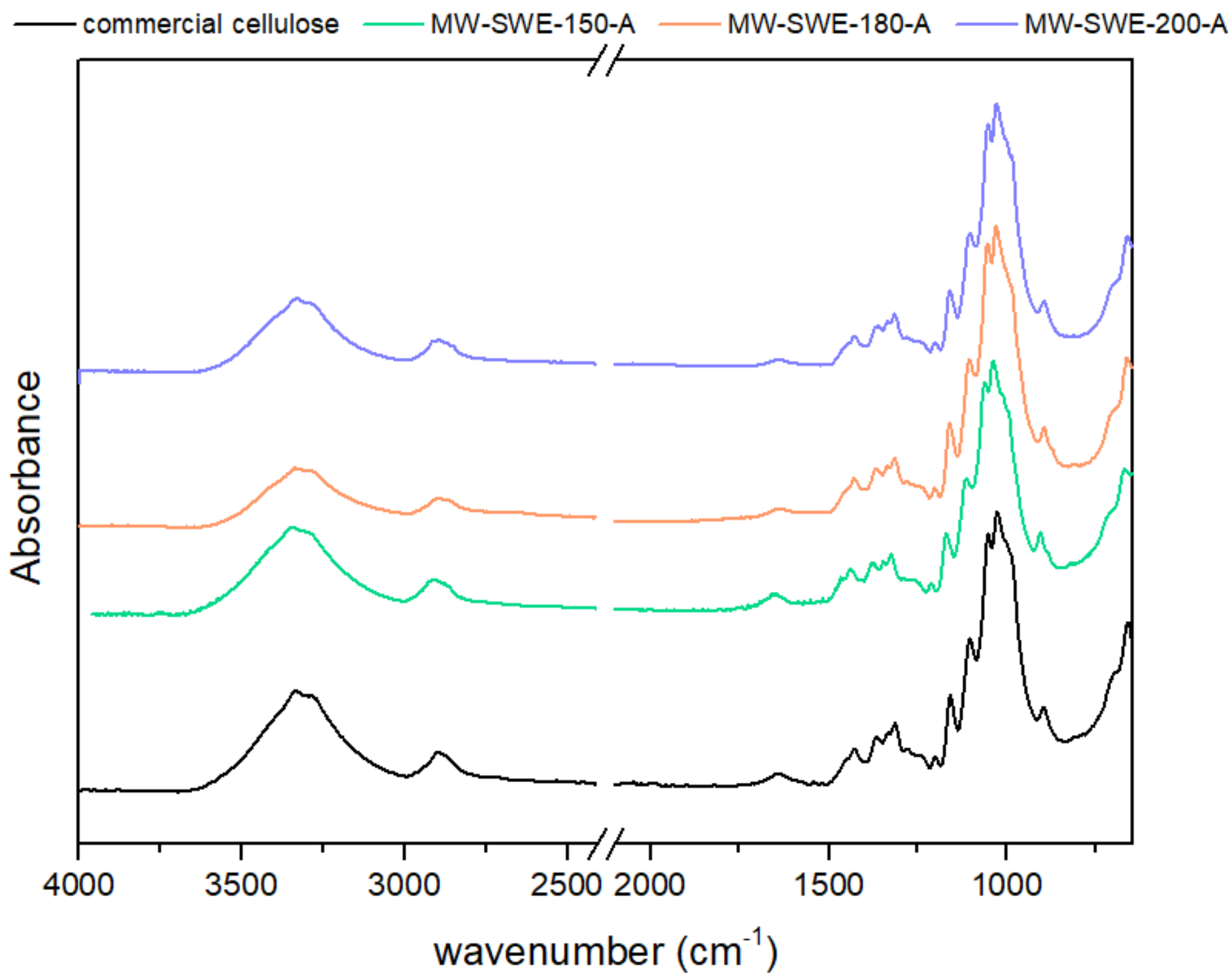

Figure S4. ATR-FTIR of commercial cellulose and post-MW-NaOH treatment biomasses

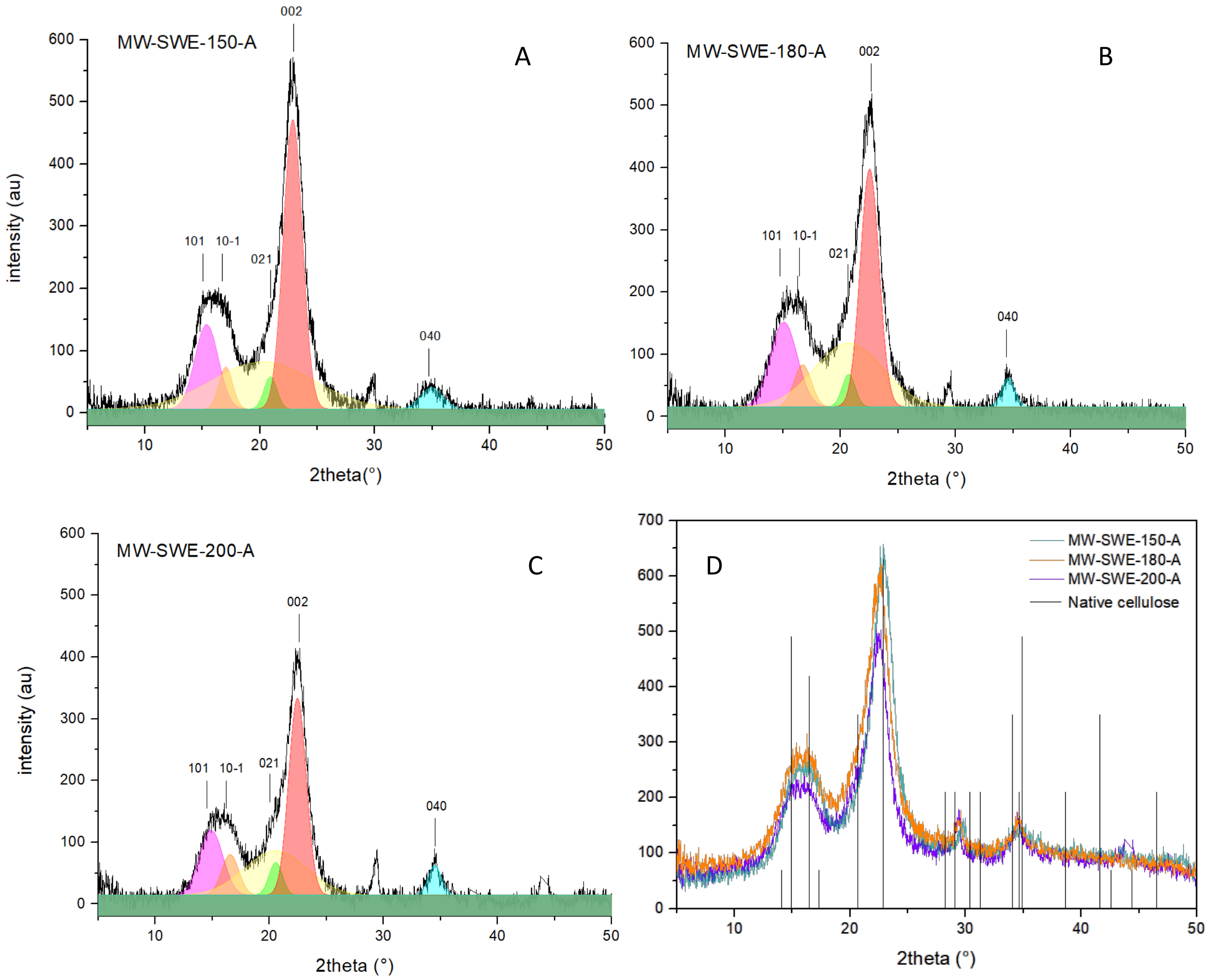

Figure S5. XRD pattern of prepared samples with deconvolution results for A) MW-SWE-150-A, B) MW-SWE-180-A, and C) MW-SWE-200-A. D) comparison of XRD patterns of prepared samples with native cellulose reference (00-003-0289)

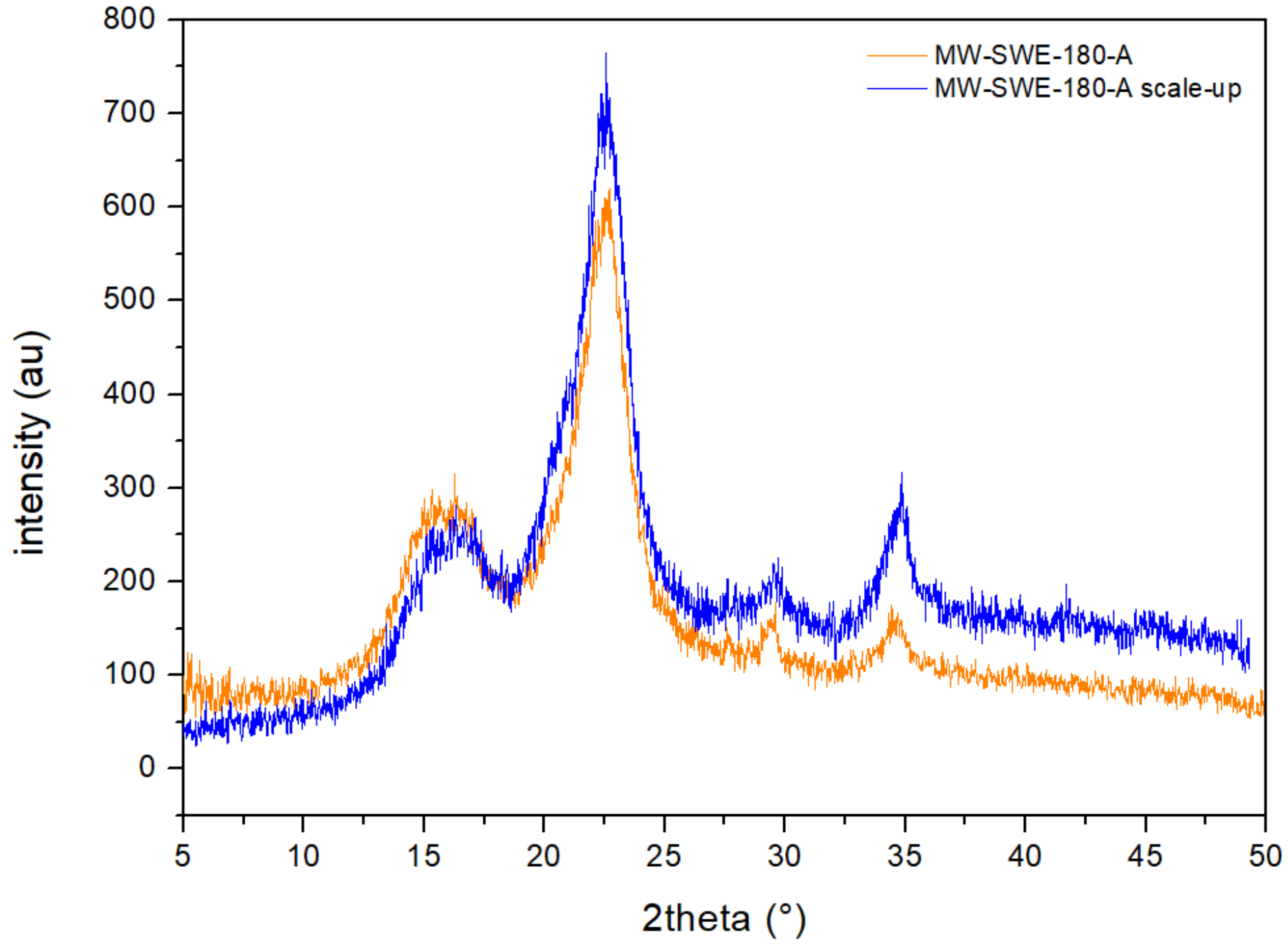

Figure S6 – XRD diffractograms of MW-SWE-180-A and scaled-up MW-SWE-180-A samples

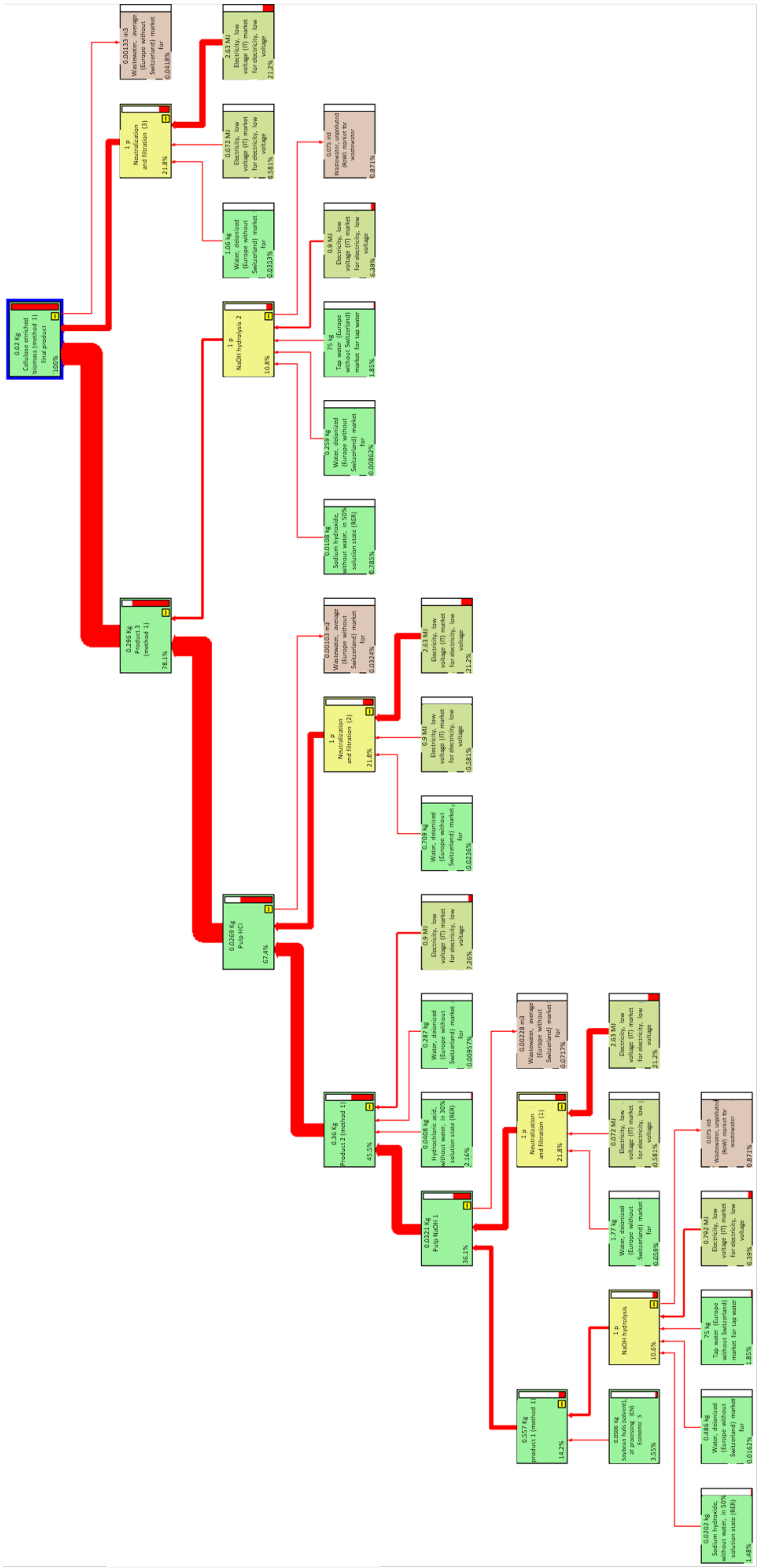

Figure S7. Sankey diagram of multi-hydrolysis cellulose extraction – CML-IA baseline V3,10 / EU25

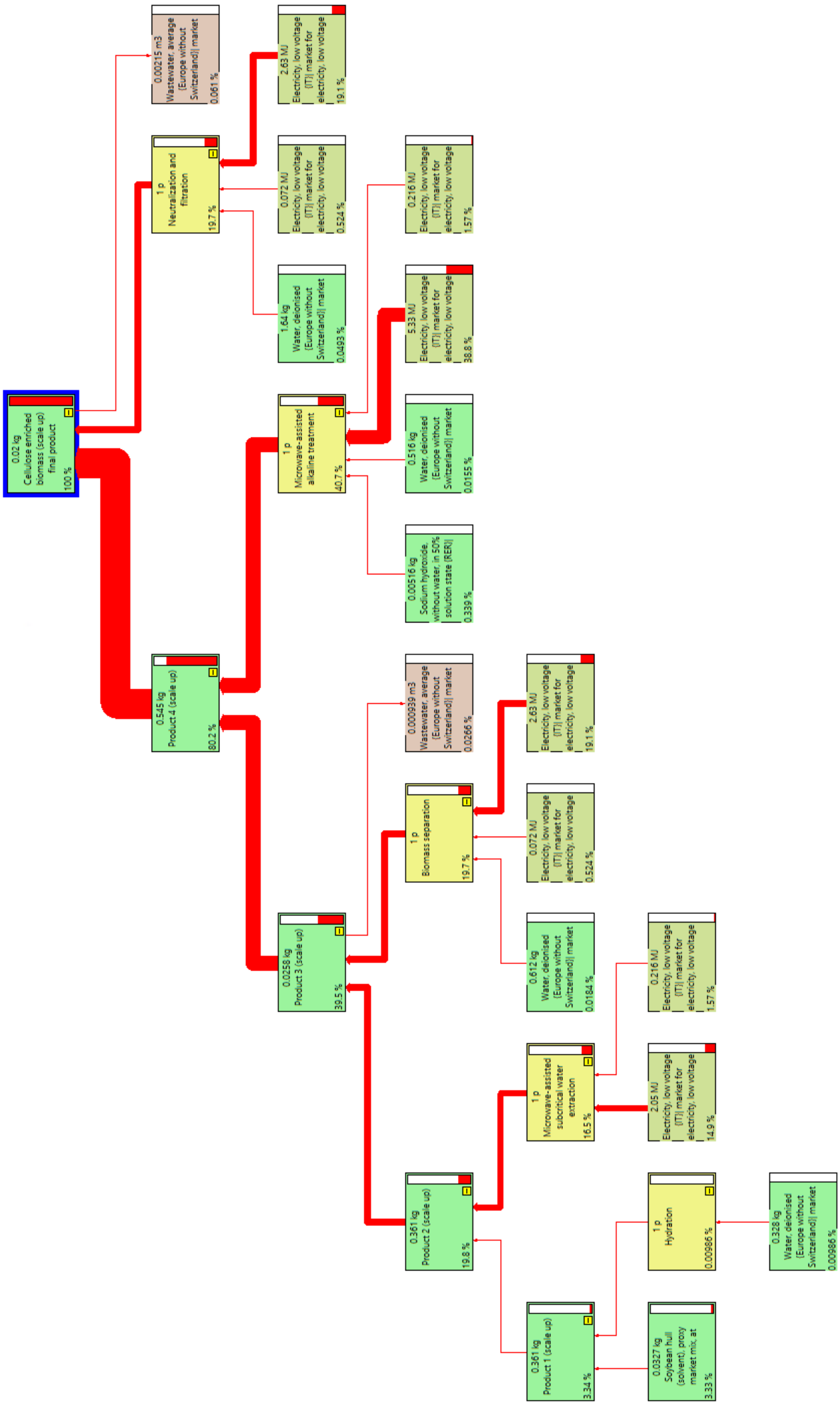

Figure S8. Sankey diagram of MW-SWE-180-A scale up - CML-IA baseline V3,10 / EU25

Table S1. TGA data related to thermal decomposition temperatures and final residues. The temperature of 750 °C was chosen as the maximum temperature reached by the samples during the ramp set at 800 °C.

Sample name	Temperature [°C]					TGA residue % at 750 °C
	20% weight loss	50% weight loss	80% weight loss	Onset	DTG peak	
MW-SWE-150	288	332	\	285	334	22.6%
MW-SWE-180	294	331	502	289	335	18.2%
MW-SWE-200	316	340	348	310	340	15.5%
MW-SWE-150-A	314	344	424	318	348	15.8%
MW-SWE-180-A	322	348	371	324	353	11.3%
MW-SWE-200-A	322	342	357	318	344	11.3%
Cellulose from multi-hydrolysis	328	357	398	325	362	13.8%
Fibrous commercial cellulose (Merck)	332	346	358	329	348	7%

Table S2. I_{002} and I_{AM} features, FMHW and calculated Crystallinity Index

Sample	I_{002}			I_{AM}		C.I. $\%_{Segal}$	C.I.$\%_{area}$
	Center (°)	Intensity	FMHW (°)	Center (°)	Intensity		
MW-SWE-150-A	22.7	616	2.25	19	150	75.6%	77.4%
MW-SWE-180-A	22.6	582	2.21	18.8	187	67.8%	72.7%
MW-SWE-200-A	22.5	484	2.02	18.4	149	69.2%	61.45%
MW-SWE-180-A scale up	22.6	696	2.34	18.5	199	71.4%	73.44%

Table S3. LCA results: CML-IA baseline V3,10 / EU25 method - Characterization for multi-hydrolysis and MW-SWE-180-A scale up.

Impact Category	Abbreviation	Unit	Multi-hydrolysis	MW-SWE-180-A scale up
**Abiotic depletion**	ADP_e	$kg_{Sbeq.}$	1,45E-05	1,64E-05
**Abiotic depletion (fossil fuels)**	ADP_f	MJ	16,09	18,10
**Global warming (GWP100a)**	GWP	$kg_{CO2eq.}$	1,213	1,345
**Ozone layer depletion**	ODP	$kg_{CFC\text{-}11eq.}$	2,87E-08	2,64E-08
**Human toxicity**	HTP	$kg_{1,4\text{-}DBeq.}$	4,291	3,496
**Freshwater aquatic ecotoxicity**	FAETP	$kg_{1,4\text{-}DBeq.}$	1,335	1,356
**Marine aquatic ecotoxicity**	MAETP	$kg_{1,4\text{-}DBeq.}$	2192	1973
**Terrestrial ecotoxicity**	TETP	$kg_{1,4\text{-}DBeq.}$	0,0305	0,0204
**Photochemical oxidation**	POCP	$kg_{C2H4eq.}$	2,54E-04	2,74E-04
**Acidification**	AP	$kg_{SO2eq.}$	4,38E-04	4,70E-04
**Eutrophication**	EP	$kg_{PO4eq.}$	1,47E-04	1,37E-04

Table S4: Comparison of literature extraction methodologies

starting biomass	Untreated biomass composition	method	conditions	cellulose %	DOI
wetland reed grass	Cellulose: 36 wt% Hemicellulose: 29 wt% Lignin: 14.6 wt%,	**STEP 1**: autoclave subcritical water hydrolysis (SbCW) **STEP 2**: alkaline pulping **STEP 3**: alkaline H_2O_2 bleaching	**SbCW**: 170 °C for 30 min at 3.5 Mpa; solid:liquid ratio 1:50. **Alkaline pulping:** 0.5M NaOH (solid-to-liquid ratio of 1:30) in a conical flask with a continuous stirring at 250 rpm at 55 °C for 1.5 h. **H_2O_2 bleaching**: 0.5-3% H_2O_2 in 0.5-0.1 M NaOH same experimental conditions followed for the pulping process.	Yield: 35.1%; Purity: 99.5 wt%	https://doi.org/10.1016/j.fuel.2021.122618
sesame hulls		**STEP 1:** A) high pressure subcritical water or B) alkaline treatment **STEP 2**: H_2O_2 bleaching **STEP 3:** acid treatment	**A) Subcritical water extraction:** solid-to-liquid ratio of 1:15 (g/mL) in a high-pressure reactor for 30 min at 180°C or **B) Alkaline treatment:** NaOH solution (4 wt%), 80 °C for 2 h with continuous magnetic stirring. **Bleaching:** 3% (w/v) H_2O_2 at 80 °C for 90 min; solid: liquid ratio of 1:50 **Acid treatment:** H_2SO_4 (55 wt%) 90 min at 45 °C; solid: liquid ratio of 1:30 (g/mL) + dyalysis	Yield: A) 11.6% B) 10.1%	10.1016/j.arabjc.2021.103178
beer bagasse	cellulose (12–25%), hemicellulose (20–25%), lignin (10–28%), starch (2–7%), proteins (20%), lipids (6–10%), minerals (2–5%); polyphenols (0.7–2%)	**STEP 1:** defatting **STEP 2**: high pressure subcritical water extraction (SWE) **STEP 3:** 4 H_2O_2 bleaching cycles	**Defatting:** petroleum ether for 8 h at 60 °C, with stirring using a solid:liquid ratio 1:4 then washed with pure solvent and filtered. **SWE:** ratio solids-water of 1:8 using a pressure reactor 150°C-4.5 bar 50 rpm 30 min. **Bleaching:** H_2O_2 4% wt% solvent-solid ratio of 30:1 at 40 °C for 1 h in four consecutive cycles, filtering and washing the sample with distilled water after each cycle.	Yield: 14%, purity: 70%	https://doi.org/10.3390/molecules29204897
rice straw	Cellulose: 36.7 % Lignin: 21.2 % Hemicellulose: 19.3 %	**STEP 1**: high pressure subcritical water extraction (SWE) **STEP 2:** 4 alkaline H_2O_2 bleaching cycles	**SWE:** 180 °C, 11 bar, 150 rpm for 30 min in a pressure reactor with solid: distilled water ratio of 1:10 (w/w). **Bleaching:** H2O2 4% pH12 was mixed at a 1:30 (w/v) ratio and heated in a water bath at 40 °C for 1h	Yield 34.90%, purity 86.2%	https://doi.org/10.1016/j.carpta.2024.100491
Posidonia oceanica waste	Cellulose: 29.6 ± 0.7% Hemicellulose: 7.9 ± 0.4% Lignin 14.3 ± 0.3% Ashes 14.3 ± 0.3%	**STEP 1:** high pressure subcritical water extraction (SWE) **STEP 2:** 4 alkaline H_2O_2 of bleaching cycles	**SWE**: solid: distilled water ratio of 1:15 (w/v), 170 °C (9.5 bar), 150 rpm, for 30 min. **Bleaching:** H_2O_2 at 4% and pH 12; solution-solid ratio of 30:1 at 40°C for 1h	Yield: 25% Purity: 85 %	https://doi.org/10.1016/j.carpta.2024.100550
Hardwood waste fine fibers	Cellulose: 57.8% Pentosan: 14.8% Lignin: 27.4%	**STEP 1**: Microwave-assisted formic acid (MAFA) treatment **STEP 2:** acidic sodium chlorite bleaching	**MAFA**: 88% formic acid, 100 °C, 4+4 h in a microwave oven. **Bleaching:** 130 mL distilled water, 1.0 mL acetic acid (99.7%), and 1.5 g sodium chlorite (80%); 75 °C in a water bath for 1 h	purity 93.2%	https://doi.org/10.1007/s10570-019-02516-8

Eucalyptus baste	Cellulose: 62.1%<br>Lignin: 25.6 %<br>Hemicellulose: 12.3 %<br>Extractives: 6.5 %	**STEP 1**: Microwave assisted mercerization<br>**STEP 2**: microwave assisted bleaching	**Mercerization:**<br>Solid:liquid ratio 1:25 (g/mL); NaOH 2.5 M; 30 min; 400 W; 100°C<br>**Bleaching:**<br>2h with 400 W (H_2O_2 32% v/v)	Yield: 62%<br>Purity: 99.1%	https://doi.org/10.1007/s10570-020-03019-7
Wheat Straw	Cellulose: 44.81±0.67%<br>Hemicellulose: 34.11±0.75%<br>Lignin: 8.75±0.31%	**STEP 1**: steam explosion<br>**STEP 2:** acid hydrolysis<br>**STEP 3**: microwave assisted alkaline hydrolysis (MW-ALK)<br>**STEP 4**: micro fluidization	**Steam explosion:**<br>100 °C; 3.0 MPa; 2 min.<br>**Acid hydrolysis:**<br>1 M HCl solution; solid–liquid ratio 1:20; 80 ± 1 °C for 2 h<br>**MW-ALK:**<br>1.5:30 solid:liquid ratio (g/mL); 2% NaOH aqueous solution; 140 ± 2 °C within 3 min and maintained for 20 min; maximum power setting 1200 W.<br>**Micro fluidization:**<br>A high-pressure microfluidizer was used to isolate CNFs under pressures of 150 MPa (21 750 psi) to 159 MPa (23 000 psi)	Yield: 23.97±2.03%<br>Purity: 94.04%	https://doi.org/10.1021/acssuschemeng.7b01108
Date Palm Biomass		**STEP 1**: Microwave assisted alkaline pretreatment (MW-pre)<br>**STEP 2:** microwave assisted bleaching (MW-BL)<br>**STEP 3**: microwave assisted NADES extraction (MW-NADES)	**MW-pre:**<br>1:30 (g/mL) solid:liquid ratio (NaOH, 5% w/v); 10 min at 90 °C.<br>**MW-BL:**<br>30% H_2O_2 v/v (pH 5.9), 10 min at 65 °C.<br>**MW-NADES:**<br>0.43 g/mL NaDES (1:1 molar ratio of choline chloride-to-citric acid anhydrate) in 1M H_2SO_4 at 90 °C for 10 min.	Yield 89%	https://doi.org/10.3390/app14209583

References

(1) Valle, C.; Grillo, G.; Calcio Gaudino, E.; Ponsetto, P.; Mazzoli, R.; Bonavita, G.; Vitale, P.; Pessione, E.; Garcia-Moruno, E.; Costantini, A.; Cravotto, G.; Tabasso, S. Grape Stalks Valorization towards Circular Economy: A Cascade Biorefinery Strategy. *ChemSusChem* **2025**, *18* (10), e202402536. https://doi.org/10.1002/cssc.202402536.

(2) Cravotto, C.; Grillo, G.; Binello, A.; Gallina, L.; Olivares-Vicente, M.; Herranz-López, M.; Micol, V.; Barrajón-Catalán, E.; Cravotto, G. Bioactive Antioxidant Compounds from Chestnut Peels through Semi-Industrial Subcritical Water Extraction. *Antioxidants* **2022**, *11* (5), 988. https://doi.org/10.3390/antiox11050988.

(3) Rojas, M. J.; Siqueira, P. F.; Miranda, L. C.; Tardioli, P. W.; Giordano, R. L. C. Sequential Proteolysis and Cellulolytic Hydrolysis of Soybean Hulls for Oligopeptides and Ethanol Production. *Ind. Crops Prod.* **2014**, *61*, 202–210. https://doi.org/10.1016/j.indcrop.2014.07.002.

(4) Pradyawong, S.; Shrestha, R.; Li, P.; Sun, X. S.; Wang, D. Effect of pH and pH-Shifting on Lignin–Protein Interaction and Properties of Lignin-Protein Polymers. *J. Polym. Environ.* **2022**, *30* (5), 1908–1919. https://doi.org/10.1007/s10924-021-02319-8.
